# Demographic surveillance over 12 years helps elicit determinants of low birth weights in India

**DOI:** 10.1371/journal.pone.0218587

**Published:** 2019-07-10

**Authors:** Aditi Apte, Rutuja Patil, Pallavi Lele, Bharat Choudhari, Tathagata Bhattacharjee, Ashish Bavdekar, Sanjay Juvekar

**Affiliations:** 1 Vadu Rural Health Program, KEM Hospital Research Centre, Pune, Maharashtra, India; 2 INDEPTH Network, East Legon, Accra, Ghana; Public Health Foundation of India, INDIA

## Abstract

**Background:**

Low birth weight is an important predictor of maternal and child health. Birth weight is likely to be affected by maternal health, socioeconomic status and quality of health care facilities.

**Objective:**

To assess trends in the birth weight, the proportion of low birth weight, maternal factors and health care facilities for delivery in villages of Western Maharashtra from the year 2004 to 2016 and to analyze factors associated with low birth weight for total birth data of 2004–2016.

**Methods:**

Data collected for 19244 births from 22 villages in Vadu Health and Demographic Surveillance System (HDSS), Pune, Maharashtra, India from the year 2004 to 2016 were used for this analysis.

**Results:**

There was an overall increase in the annual mean birth weight from 2640.12 gram [95% CI 2602.21–2686.84] in the year 2004 to 2781.19 gram [95% CI 2749.49–2797.95] in the year 2016. There was no secular trend to show increase or decrease in the proportion of low weight at birth. Increasing maternal age (>18 years) compounded with better education, reduced parity and increasing number of institutional deliveries were significant trends observed during the past decade. Low birth weight was found to be associated with female gender, first birth order, poor maternal education and occupation as cultivation.

**Conclusion:**

Changes in maternal age, education, occupation, and increased institutionalized deliveries contributed in to increasing birth weights in rural Maharashtra. Female gender, first birth order, poor maternal education and occupation of cultivation are associated with increased risk of low birth weight.

## Introduction

Low birth weight (LBW) is defined by the World Health Organization as birth weight of less than 2500 grams (gm). LBW is the single most important cause of neonatal mortality[1][2]. A high incidence of LBW is not only associated with increased perinatal morbidity and mortality but can also be associated with increased risk of lifestyle illness at later stages of life e.g. diabetes mellitus, hypertension[3]. Low birth weight for gestation is one of the leading causes for the global burden of disease (third position for men and fourth position for women) in the year 2016. This is despite a reduction in daily adjusted life years (DALY) and age-standardized DALY rates since the year 1990 [4].

In the year 2013, the global incidence of LBW was 16%, whereas in South Asia it was found to be 28% [5]. The proportion of LBW in India has reduced from 25.2% in National Family Health Survey (NFHS)-1 (Year 1991–92) to 18% in NFHS-4 (Year 2015–16) [6] but is still high as compared to many developed nations in the world [7].

There is strong evidence to show that the incidence of LBW is affected by multiple factors including genetic, placental, fetal, maternal and social factors [8][9][10]. Maternal factors such as maternal age, low pre-pregnancy body mass index, poor gestational weight gain, short stature and maternal comorbidities especially anemia are known to increase the risk of LBW[8][11]. The nutritional status of an expectant mother is also influenced by socioeconomic status. The socioeconomic status is, in turn, dependent upon the educational status of the family members and is likely to influence access to health care. Sex of the child, high parity, too short (<18 months) or too long (>59 months) interval between two pregnancies are some biological factors associated with LBW[12][13].

Although the district and national family health surveys provide statistics of important child health-related indicators, they do not provide a longitudinal analysis of health indicators. Lack of extensive longitudinal community-based studies on birth-related indicators in India makes it difficult to assess trends in birth weights and birth-related indicators[14][15]. Health and Demographic Surveillance Systems (HDSS) provide a unique opportunity for systematic data collection of birth, death and other health events of a well-defined population in a periodic manner[16].

The present paper is an analysis of 19244 births captured as part of Vadu HDSS over a period of 12 years (2004–2016). In this paper, we summarize trends in birth weight, proportion of LBW, maternal factors (age, occupation, education, parity) and health care facilities for delivery (place of delivery and personnel conducting delivery) in Vadu HDSS. Secondly, we have analyzed factors associated with LBW in Vadu HDSS.

## Methods

### Ethical consideration

The Vadu HDSS has received approval from the Institutional Ethics Committee of KEM Hospital Research Centre, Pune (KEMHRC EC Ref No: KEMHRC/VSP/Dir.Off/EC1371). This being a demographic surveillance, a verbal informed consent was taken from an adult member of the household, preferably head of the household during their registration into HDSS [17][18] and during each rounds of data collection thereafter. Moreover, community permission was obtained from village heads during community meetings before initiating data collection from their respective villages.

### The population

We used birth data collected from all the births in Vadu HDSS from the year 2004 to 2016. The Vadu HDSS under the auspices of KEM Hospital Research Centre Pune (KEMHRC) longitudinally monitors a rural population of 171462 (as on December 2016) residing in 22 villages. The analysis was conducted for babies of mothers who were usual residents of Vadu. Data of mother-baby diads who visited Vadu only during childbirth were excluded from this analysis.

Vadu has a predominantly agrarian economy. It is a homogenous population in terms of religion and lifestyle. In the last five years, some villages have experienced rapid industrialization. This has resulted in increased land prices which led to a drastic change in the economic condition of landowners who have sold their lands for non-agricultural purpose. The industrialization has provided avenues for newer occupational opportunities [19][20]. Health facilities include one rural hospital and a primary health care center in the government sector. The private sector dominates with a little over 100 health care facilities including either inpatient and/ or outpatient facilities.

### Data collection

The Vadu HDSS captured data using standard data collection tools widely used by several demographic surveillance sites across the globe. Trained Field Research Assistants (FRA) conducted the house to house surveys biannually using standardized structured interview schedule as a tool for birth, death, marriage, pregnancy, and migration. The respondents included adult family members from each household who were approached for interviews followed by a verbal consent[21][22][23]. The interview data were captured by FRAs in the field, using paper forms in the initial years. Since 2010, the data were directly captured in electronic forms laptops or android tablet (S1 File). The FRAs were supervised by Field Research Supervisors (FRS) on a daily basis.

Data on birth weight were based on information using hospital records, vaccination cards or maternal recall. Hospital records were used as the primary source of data; when not available, information based on vaccination card and/or maternal recall was used in this order. Data of all live births reported from the year 2004 till 2016 have been included in this analysis. Apart from birth weight, the following data were extracted from the HDSS database: sex of the baby, birth order, maternal age, maternal occupation, maternal education, place of delivery and health professional conducting the delivery. The data were independently extracted by the database management team from KEMHRC, Vadu.

The data underwent dual quality control—quality control related to data collection and quality control related to data management. As a part of the quality control, a team of supervisory cadre (the FRS) repeated data collection for random 10% households. The FRSs also accompanied the 10% randomly selected FRAs on the field at the time of data collection. For the quality control related to data management, there were set of algorithms and programs which were pre-tested and were run on data at regular intervals.

### Variables

The outcome of birth weight was assessed by calculating mean birth weight and the proportion of LBW infants for each year. This included babies that were preterm as well as small for gestational age. The explanatory variables included infant sex, birth order, maternal age, maternal education, occupation, place of delivery and health professional conducting the delivery. These variables were extracted from the Vadu HDSS database. The clean and fully documented datasets from Vadu HDSS are regularly published online as a part of the INDEPTH Data Repository[24].

### Data analysis

To address the first objective, we compared the change in mean birth weight and proportion of LBW amongst all live born babies annually from the year 2004 till 2016. We compared the change in birth weight at each quartile over time. Moreover, we assessed trends in birth characteristics such as parity, place of delivery, maternal age, education, and occupation. Birth weight and maternal age were expressed as mean and standard deviation. All other variables were categorical and were expressed as proportions. Regression analysis was used to analyze the trends in continuous data and Pearson’s Chi-square test was used to analyze the trend in the categorical variables.

To investigate the association between LBW and the explanatory variables; we used logistic regression analysis on total live birth data from the year 2004 to 2016. Crude odds ratios (ORs) with 95% confidence intervals were estimated for all the explanatory variables. Adjusted ORs were calculated by entering all the significant explanatory variables including the year of birth into the logistic regression model. Each variable entered the model if the likelihood-ratio test for its coefficient was significant (P<0.05). The statistical analyses were carried out using STATA v11 software.

## Results

### Births

A total of 24,116 live babies were born from the year 2004 to 2016 after excluding missing values for gender and maternal indicators and 110 still births from data of 24,363 births. Data of 19,244 babies born to resident mothers in Vadu HDSS from the year 2004 to 2016 were considered for this analysis.

**Fig 1:**
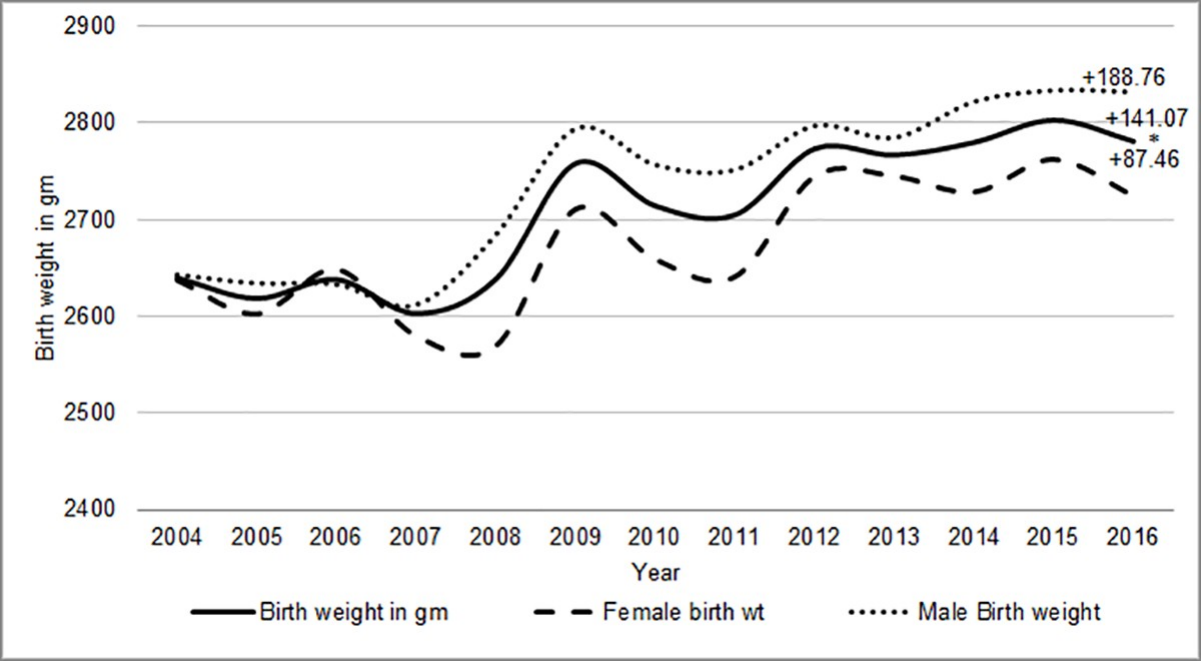
Distribution of mean birth weights from the year 2004 to 2016. *p for trend using regression <0.001.

**Fig 2:**
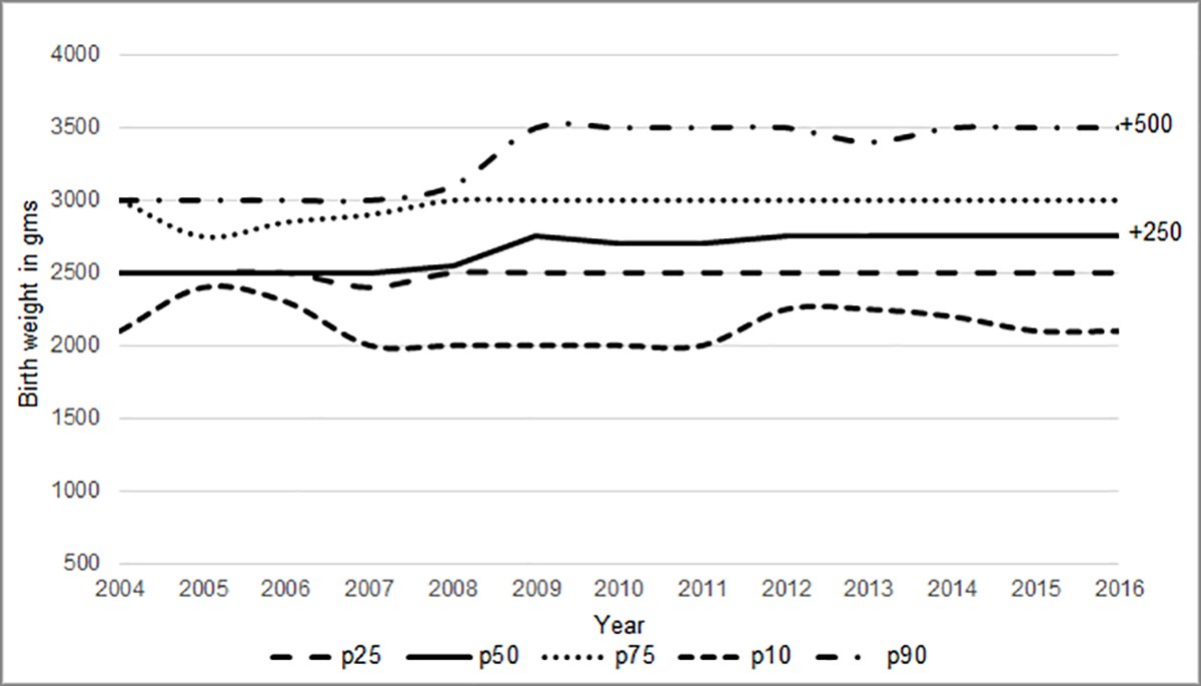
Trends in different quartiles of birth weights. p10-10^th^ percentile, p25-25^th^ percentile, p50-median, p75- 75^th^ percentile, p90-90^th^ percentile.

The overall mean birth weight was 2742.56 ± 507.84 gm. There was an increase in the annual mean birth weight from 2640.12 gm [95%CI 2602.21–2686.84] in the year 2004 to 2781.19 gm [95% CI 2749.49–2797.95] in the year 2016 [p for trend using regression <0.001]. The male babies were heavier than the female babies [mean wt-2737± 83 gm vs. 2674 ±66 gm, p<0.001 using t test]. The mean birth weights for male babies increased by 188.76 gm (7.14%) and for female babies by 87.46 gm (3.32%) from the year 2004 to 2016 [Fig 1]. We compared the trend in different quartiles of birth weight. There was no shift in the 10^th^ or 25^th^ percentile of the data but there was an increase in the median and 90^th^ percentile by 250 gm and 500 gm respectively [Fig 2].

There were a total of 3176 (16.49%; 95% CI 0.16–0.17) babies with a reported birth weight of less than 2500 gm. The prevalence of LBW was significantly higher in females as compared to males [17.57% (95% CI-16.58–18.38) vs. 15.56% (95% CI-14.86–16.27); p<0.001].Summary characteristics of the total population of low birth weight are: Mean 1985.76 gm, SD 327.87 gm, Median 2000 gm, Minimum 1800 gm and Maximum 2490 gm. The proportion of LBW infants varied from 10.34% to 22.45% across the duration and there was no secular trend towards an increase or decrease in the proportion of LBW infants [Table 1]. The highest percentage of LBW was reported in the year 2008 [23.69%] and decreased since then by 32% with 15.96% of LBW babies reported in the year 2016.

**Table 1:** Trends in birth characteristics from 2004 to 2016.

Birth characteristics		2004 (N = 400)	2005 (N = 455)	2006 (N = 115)	2007 (N = 234)	2008 (N = 660)	2009 (N = 1255)	2010 (N = 972)	2011 (N = 1799)	2012 (N = 2929)	2013 (N = 2098)	2014 (N = 2922)	2015 (N = 3667)	2016 (N = 1738)
Low Birth weight*,n(%)		51(12.73)	47(10.34)	14(12.32)	54(23.03)	156(23.67)	205(16.35)	218(22.45)	381(21.20)	427(14.57)	269(12.83)	440(15.05)	600(16.37)	277(15.96
Birth Order*, n(%)	First	170(42.44)	196(43.10)	56(48.77)	124(53.11)	298(45.11)	635(50.60)	489(50.30)	871(48.43)	1481(50.57)	1115(53.15)	1649(56.43)	1994(54.38)	881(50.67)
	Second	154(38.59)	186(40.89)	42(36.45)	82(34.85)	274(41.55)	482(38.42)	355(36.49)	707(39.30)	1167(39.86)	778(37.10)	983(33.65)	1302(35.50)	674(38.78)
	Third	53(13.13)	50(11.08)	13(11.33)	24(10.17)	61(9.29)	106(8.43)	97(9.98)	172(9.56)	225(7.68)	165(7.85)	233(7.99)	270(7.36)	137(7.86)
	Fourth	20(4.91)	11(2.46)	3(2.96)	3(1.24)	18(2.79)	23(1.83)	25(2.55)	37(2.05)	39(1.34)	29(1.36)	41(1.41)	69(1.88)	34(1.97)
	Fifth	4(0.93)	11(2.46)	1(0.49)	1(0.62)	8(1.26)	9(0.72)	7(0.68)	12(0.67)	16(0.54)	11(0.54)	15(0.51)	33(0.89)	13(0.72)
Place of delivery*, n(%)	Public Hospital	64(16.05)	64(14.04)	17(14.78)	61(25.93)	117(17.67)	201(16.03)	218(22.45)	368(20.46)	585(19.96)	604(28.80)	1022(34.97)	1386(37.81)	734(42.23)
	Private Hospital	267(66.71)	266(58.37)	74(64.53)	159(68.05)	483(73.18)	970(77.29)	656(67.49)	1262(70.17)	2228(76.05)	1431(68.21)	1803(61.69)	2078(56.66)	929(53.45)
	Home	40(10.08)	47(10.34)	10(8.37)	11(4.56)	59(8.94)	79(6.32)	91(9.38)	158(8.80)	108(3.70)	56(2.68)	78(2.66)	160(4.36)	65(3.74)
	Other	29(7.16)	78(17.24)	14(12.32)	3(1.24)	1(0.21)	5(0.36)	7(0.68)	10(0.58)	8(0.28)	7(0.32)	20(0.67)	43(1.17)	10(0.58)
Type of birth attendant*, n(%)	Doctor	308(77.06)	327(71.80)	86(74.88)	160(68.46)	409(62.01)	1091(86.95)	774(79.65)	1531(85.11)	2572(87.82)	1902(90.66)	2461(84.22)	1721(46.94)	326(18.74)
	Nurse	22(5.44)	18(3.94)	5(3.94)	29(12.45)	144(21.86)	92(7.32)	123(12.61)	126(6.99)	250(8.55)	128(6.12)	335(11.45)	1778(48.49)	1345(77.37)
	Trained birth attendant	20(5.04)	25(5.54)	11(9.36)	41(17.43)	78(11.87)	23(1.87)	7(0.68)	16(0.90)	33(1.12)	34(1.63)	72(2.47)	27(0.74)	16(0.91)
	Untrained birth attendant	19(4.64)	21(4.56)	2(1.97)	1(0.41)	19(2.86)	24(1.91)	34(3.53)	39(2.19)	35(1.18)	20(0.95)	26(0.90)	56(1.52)	11(0.62)
	Other	31(7.82)	64(14.16)	11(9.85)	3(1.24)	9(1.40)	24(1.95)	34(3.53)	86(4.80)	39(1.34)	13(0.63)	28(0.96)	85(2.31)	41(2.35)

*p<0.001 using Pearson's chi-square test; N indicates total number of data points available for analysis for the given year

### Parity

There was a decrease in the parity from the year 2004 to 2016 for particularly babies born with birth order of 3 or more [Table 1].

### Health care facilities for birth

There was a decrease in the proportion of births at private health care setup from the year 2012 onwards whereas the deliveries conducted at public health set-ups steadily increased from the year 2012 to 2016. Overall, the proportion of institutional deliveries increased to a great extent during the 12 years timespan and the number of home deliveries reduced to approx. 4% by 2016 [Table 1].

In parallel with the trend of institutional deliveries, there was an increase in the proportion of deliveries conducted by trained health care professionals (doctor, nurse or trained birth attendant) from the year 2004 to 2016. After the year 2013, there was an increasing trend in the number of deliveries conducted by nurses as compared to doctors [Table 1].

### Maternal characteristics

#### Maternal age

The average maternal age increased from the year 2004 to 2016 which but was not statistically significant [p = 0.2586] [Table 2]. Proportion of mothers with age < 18 years decreased from 7.27% in the year 2004 to 2.83% in the year 2016 [p<0.001]. The proportion of mothers with age >35 years at childbirth was 1–2% throughout the time period [p = 0.926].

**Table 2:** Trends in maternal characteristics from 2004 to 2016.

Maternal characteristic	2004	2005	2006	2007	2008	2009	2010	2011	2012	2013	2014	2015	2016
	(N = 400)	(N = 455)	(N = 115)	(N = 234)	(N = 660)	(N = 1255)	(N = 972)	(N = 1799)	(N = 2929)	(N = 2098)	(N = 2922)	(N = 3667)	(N = 1738)
**Maternal age at child birth in years#**													
Mean	22.26	22.83	22.92	21.73	22.62	22.81	23.08	23.27	23.35	23.57	22.77	23.78	23.88
SD	4.91	4.24	4.54	5.11	4.31	4.17	3.89	3.86	3.82	3.81	3.78	3.84	4.37
Maternal Occupation *													
Homemaker, n(%)	278(69.44)	255(56.00)	70(60.78)	130(55.40)	442(67.00)	994(79.18)	811(83.45)	1502(83.48)	2521(86.08)	1836(87.53)	2521(86.29)	3319(90.50)	1582(91.05)
Cultivator, n(%)	108(26.94)	184(40.50)	39(34.31)	96(40.85)	200(30.36)	228(18.17)	129(13.31)	255(14.16)	345(11.79)	176(8.41)	229(7.84)	135(3.69)	48(2.76)
Industry, n(%)	14(3.61)	15(3.25)	5(3.92)	8(3.29)	16(2.48)	32(2.56)	31(3.24)	41(2.30)	60(2.05)	34(1.63)	56(1.93)	69(1.89)	51(2.94)
Other, n(%)	—	1(0.25)	1(0.98)	1(0.47)	1(0.17)	1(0.09)	—	1(0.06)	2(0.07)	48(2.28)	111(3.79)	140(3.81)	49(2.82)
Student, n(%)	—	—	—	—	—	—	—	—	—	3(0.16)	4(0.15)	4(0.12)	7(0.43)
Maternal Education*													
Illiterate mothers, n(%)	19(4.76)	18(3.98)	6(5.36)	18(7.69)	31(4.68)	70(5.59)	81(8.32)	112(6.22)	128(4.36)	87(4.17)	117(3.99)	217(5.85)	75(4.34)
Completion of class 4, n(%)	172(43.11)	219(48.23)	47(41.07)	118(50.43)	306(46.37)	511(40.71)	387(39.81)	718(39.92)	998(34.07)	661(31.51)	917(31.38)	1071(29.21)	499(28.73)
Completion of class 10, n(%)	177(44.36)	195(42.92)	53(46.43)	88(37.61)	294(44.56)	565(45.04)	429(44.18)	826(45.91)	1507(51.44)	1069(50.94)	1505(51.52)	1845(50.32)	868(49.94)
Graduate, n(%)	26(6.52)	16(3.54)	7(6.25)	6(2.56)	20(3.02)	89(7.09)	62(6.34)	114(6.33)	248(8.45)	214(10.22)	306(10.46)	422(11.51)	236(13.58)
Postgraduate, n(%)	5(1.25)	6(1.33)	1(0.89)	4(1.71)	9(1.36)	20(1.57)	13(1.35)	29(1.62)	49(1.68)	67(3.17)	78(2.66)	114(3.11)	59(3.41)

# p = 0.2586 using ANOVA

*p<0.001 using Pearson's chi-square test;; N indicates total number of data points available for analysis for the given year

#### Maternal occupation

Cultivation and homemaking were the two major maternal occupations over the 13 year period. There was a reduction in the number of women cultivators and an increase in the number of homemakers. These observations tally with a large number of agricultural lands being sold to real estate developers in the last decade. Amongst the total observations, the majority of the mothers were homemakers irrespective of their education levels. Amongst the cultivators, 5% of mothers were illiterate, 43.14% had primary education and 47.9% had secondary education.

#### Maternal education

There was no secular trend in the proportion of illiterate mothers over the year 2004 to 2016 [Table 3], however more mothers were found to achieve a secondary or higher level of education after the year 2010 as compared before 2010. More than 96% of the educated mothers (with at least completion of 10^th^ class) had institutional deliveries. Of all the illiterate mothers, 77.30% delivered in hospitals and 20.45% had home deliveries. The illiterate mothers contributed to the maximum number of home deliveries across all maternal education categories. Moreover, 73–85% of the babies with birth order of 3, 4 and 5 or more were born to mothers either illiterate or with primary education.

**Table 3:** Factors associated with low birth weight based on logistic regression.

Characteristic	N	Low birth weight n(%)	Crude Odds ratio (95%CI)	Adjusted Odds ratio (95% CI)
*Sex*				
Male	10447	1628(15.58)	Referent	Referent
Female	8797	1548(17.6)	1.16[1.07–1.25]	1.16[1.08–1.27]
*Birth Order*				
First	9839	1653(16.8)	1.09[1.00–1.18]	1.13[1.03–1.24]
Second	7257	1130(15.57)	Referent	Referent
Third	1637	290 (17.71)	1.16[1.00–1.33]	1.07[0.91–1.25]
Fourth	372	66 (17.6)	1.15[0.87–1.52]	1.00[0.74–1.35]
Fifth and more	138	37 (26.8)	1.98[1.35–2.91]	1.44[0.92–2.24]
*Maternal Age*				
≤ 18 years	680	133 (19.50)	1.24 [1.02–1.50]	1.18[0.95–1.47]
>18-≤ 30 years	17483	2852 (16.31)	Referent	Referent
>30-≤ 35 years	824	137 (16.54)	1.01[0.84–1.22]	1.03[0.84–1.27]
> 35 years	257	54(21.01)	1.36[1.00–1.84]	1.25[0.89–1.78]
*Maternal education*				
Illiterate	951	211(21.59)	1.52[1.29–1.79]	1.42[1.17–1.72]
Completion of class 4	6623	1218(17.32)	1.25[1.15–1.36]	1.21[1.11–1.33]
Completion of class 10	9436	1439(15.27)	Referent	Referent
Graduate	1771	253(14.33)	0.92[0.80–1.06]	0.91[0.78–1.07]
Postgraduate	403	54(13.50)	0.73[0.55–0.99]	0.52[0.36–0.76]
*Maternal occupation*				
Homemaker	14900	2407(16.15)	Referent	Referent
Cultivator	1985	376(18.90)	1.21[1.07–1.37]	1.15[1.02–1.31]
Industry	403	59(14.60)	0.89[0.67–1.17]	0.90[0.68–1.20]
Other	340	53(15.60)	0.97[0.72–1.30]	0.97[0.72–1.32]
Student	25	2(8.00)	0.45[0.11–1.91]	0.72[0.16–3.15]
*Place of delivery*				
Public Hospital	5417	901 (16.63)	Referent	Referent
Private Hospital	12614	2041 (16.18)	0.96[0.89–1.05]	0.99[0.90–1.09]
Home	971	198(20.39)	1.28[1.08–1.52]	1.13[0.94–1.37]
Transit	32	7(21.80)	1.40[0.61–3.25]	1.40[0.60–3.28]
Others	210	29(13.74)	0.80[0.54–1.18]	0.72[0.47–1.11]
*Year of birth*				
Till 2010	4091	761(18.60)	Referent	Referent
After 2010	15153	2412(15.91)	0.83[0.76–0.91]	0.86[0.78–0.95]

Note: maternal age, maternal education, maternal occupation, sex, birth order, place of delivery and year of birth were adjusted in the final model

### Analysis of risk factors for low birth weight

Data for total of 19244 babies born between the year 2004 to 2016 were entered in the logistic regression analysis. The crude and adjusted odd’s ratios are shown in Table 3.

In univariate analysis, female gender, maternal age of <18 years and more than 35 years, cultivation as maternal occupation, maternal illiteracy, home deliveries, birth till the year 2010 and birth order of first or third or fifth or more were found to be associated with LBW. All the proposed variables were found to be statistically significant and hence were retained in the model. In the adjusted analysis, female gender, first birth order, poor maternal education, maternal occupation, and birth until the year 2010 were associated with increased odds of having LBW. The birth order of three and five or more, maternal age <18 years and home deliveries were not significantly associated with LBW in the adjusted model.

## Discussion

This analysis depicts an increasing trend in birth weight in the rural population of Vadu HDSS over the last 12 years. There was a decreasing trend in the proportion of mothers aged less than 18 years and proportion of cultivator mothers whereas increasing trend was observed in maternal education, which is parallel to the national trend [6]. The average proportion of LBW in Vadu HDSS was lower than the national average for rural population i.e. 20.9% in the year 2004–5 and 18% in the year 2015–16 [6][7]. Two other studies from rural Maharashtra showed 37% and 33% incidence of low birth rate [25][26].

Female gender, first birth order, poor maternal education, and maternal occupation were found to be significantly associated with the risk of LBW.

Females were associated with lower birth weights than male babies and had a 16% increased risk of LBW. This is consistent with the earlier literature on LBW [27–30]. The exact reason for this gender difference is not known, however, some role of androgens secreted by fetal testes in male fetuses [31], maternal food intake[32] and vitamin D receptor genotype influencing intrauterine and early postnatal growth, through interactions with gender-related growth regulators is suggested[33].

Babies with first birth order were associated with a 13% increased risk of LBW as compared to the second birth order in the multivariate analysis. This is similar to the previous literature which showed that birth weight increases up to 3^rd^ birth order and decreases thereafter [34–36]. Both maternal age and birth order are important determinants of LBW and having first pregnancy at an adolescent (<18 yrs) or advanced (>35 yrs) age is associated with increased risk of LBW [35–37]. But in our adjusted analysis, early or advanced maternal age was not found to exert any significant effect on LBW.

Maternal health and the sociocultural environment has a significant impact on birth outcomes [10,13,38]. In our analysis, illiterate mothers and mothers with primary education (completion of 4^th^ class) were shown to have 42% and 21% increased risk of LBW as compared to mothers with secondary education (completion of 10^th^ class). This is in consensus with a meta-analysis conducted by Silverstrin, which shows that high maternal education has a protective effect against LBW [39]. The association of LBW with low maternal education has been shown in several other studies including NFHS-4[40]. This could be due to the fact that poor maternal education is usually associated with the low socioeconomic status of the families, lower weight gain during pregnancy, late start of prenatal care, and fewer antenatal consultations. Moreover, mothers with good education are more likely to have a better knowledge of health care and capable of decision making regarding their own health and care [8,41,42]. This is consistent with our findings that illiterate mothers had the highest number of home deliveries and the better the maternal education status, the higher was the proportion of institutional deliveries. Also, illiterate women were found to have high parity (3 or more) than well-educated women, which itself is a risk factor for LBW [34–36].

The association of maternal occupation with the risk of LBW has been two way. Overall employment during pregnancy has been found to be associated with reduced risk of LBW [43] but certain occupations (e.g. textile, food service) have been found to adversely increase the risk of low birth weight [44,45]. In the present analysis, the maternal occupation of cultivation was found to be associated with a 15% increased risk of LBW as compared to homemaker mothers. Agricultural women are reported to have increased risk of adverse birth outcomes. A Spanish study reported an increased risk of LBW in agricultural women suggesting exposure to pesticides as one of the reasons [46,47]. However, the association of maternal occupation with LBW is perplexed due to interactions with demographic and socioeconomic factors [44].

Children born after the year 2010 had a 14% reduced risk of LBW as compared to children born till the year 2010. The exact reasons for this improved risk of LBW are not evident. However, this analysis showed that this reduced risk of LBW is associated with an increasing trend in maternal education and an increased rate of institutional deliveries.

The year 2012 onwards, there were increased deliveries conducted at public health facilities and there was a drop in the proportion of deliveries conducted at private health care facilities. This coincides with the initiation of free ambulance services by government of Maharashtra [48] and the implementation of Janani Suraksha Yojana (JSY) by Government of India by which pregnancy registration and institutionalized deliveries in the public health sector are being promoted by providing financial incentives to families[49–51]. Although we observed an increasing trend of institutional deliveries and birth weight, the interaction between these variables is complex and the role of many more variables cannot be neglected.

The results of this analysis of surveillance data are in agreement with previous studies which showed that maternal age [35], parity [34–36], level of physical work [44,45], maternal education [39], and socioeconomic status [8,10,26,42,52] are known to be associated with the birth weight of the child.

The present paper has several limitations. The data on birth weights are based on the reported data collected by field workers from the medical records of the families, vaccination cards and parental recall and the exact distribution of data across all the data sources is not available. The outcome includes LBW irrespective of the gestational age and thus includes both premature as well as small for gestational age babies. As gestational age data were not available for all the babies, we have not included the risk of LBW due to prematurity in the analysis. Although socioeconomic status is a confounding variable for many of the variables, it could not be included in the current analysis. There are other indicators such as maternal nutritional status, gestational weight gain, antenatal visits, consumption of iron and folic acid, sanitation, consanguineous marriages which are likely to influence birth outcomes [29,30,53] which have not been taken into consideration. As HDSS follows up a selected population biannually, data on these indicators could not be included. The data from HDSS increased in numbers after the year 2012 as compared to earlier years which might be as a result of the use of electronic devices for data capture on the field since the year 2013 and an increase in the rates of immigration. Despite the urbanization trend seen in the last decade, the population under surveillance in HDSS is primarily rural and hence the effect of place of residence (rural vs urban) on the risk of low birth weight could not be assessed.

## Conclusion

The longitudinal nature of HDSS has proved to be of immense value in getting demographic trends in Vadu HDSS area. We demonstrate that increasing maternal age (>18 years), decreasing parity, improved maternal education and increased institutionalized deliveries go parallel with increasing birth weights over the study period. Female gender, first birth order, poor maternal education and maternal occupation of cultivation are associated with increased risk of low birth weight.

## Supporting information

S1 File Vadu HDSS forms for birth event and assessment of socioeconomic status. (PDF)
