# Supplementary material for: Demographic surveillance over 12 years helps elicit determinants of low birth weights in India
Source: PLoS One. 2019 Jul 10;14(7):e0218587. doi: 10.1371/journal.pone.0218587 (PMC6619655; doi:10.1371/journal.pone.0218587)
Supplement: S1 File — (PDF) [file pone.0218587.s001.pdf]

VADU HEALTH AND DEMOGRAPHIC SURVEILLANCE SYSTEM, (VADU HDSS)
VADU RURAL HEALTH PROGRAM, K.E.M HOSPITALRESEARCH CENTRE, PUNE
BIRTH EVENT FORM

Starting time of the interview: □□:□□		
Interviewer Name : ________________ Signature: __________ Date □□/□□/□□□□		
PID<br>Foetus □□□ □□□ □□□□ □□<br>Mother □□□ □□□ □□□□ □□<br>Father □□□ □□□ □□□□ □□		CID<br>Foetus □□□ □□□ □□□□ □□<br>Mother □□□ □□□ □□□□ □□<br>Father □□□ □□□ □□□□ □□
Q0001	Household Number<br>*(Record the HH No. Where the mother belongs)*	□□□ □□□ □□□□<br>Village Wadi Household Number
Q0002	Name of the head of household<br>(of above mentioned household)<br>Village Name<br>Wadi Name<br>Landmark<br>Phone Number	________<br>________<br>________<br>________<br>________
Q0003	What is the relationship of child with head of the household?	~~01 Self~~<br>~~02 Spouse *(wife/husband)*~~<br>03 Son/daughter<br>~~04 Daughter/son-in-laws~~<br>05 Grandson/daughter<br>~~06 Mother/father~~<br>~~07 Father/mother-in-laws~~<br>08 Brother/sister<br>~~09 Step wife~~<br>~~10 Grandmother/ father~~<br>11 Other relationship *(Specify)*<br>________<br>12 No relation
Q0004	Name of the Respondent *(Respondent should be head of the household or preferably the adult member of the house)*	________

Q0005	Date of Birth of the child	□□/□□/□□□□
Q0006	Type of birth	01 Live birth<br>02 Still birth*(If answer is 02 then don't ask Question -009,015,016,017,018,019)*
Q0007	Sex of the child	01 Male<br>02 Female
Q0008	Birth is registered at	01 Gram Panchayat<br>02 Corporation<br>03 Others (Specify)__________________<br>04 So far not registered
Q0009	Name of the child (do not ask for stillbirth)	____
Q0009a	Name of the mother of the child	____
Q0009b	Name of the father of the child	____
Q0010	Permanent address of child's mother (If she is from other than Vadu study area)	____
Q0011	Number of births in Present / current delivery?	01 First<br>02 Second<br>03 Third
Q0012	Place of delivery	01 Government hospital<br>02 Private hospital<br>03 Home<br>04 In transit towards hospital<br>05 Other (specify)_____________________
Q0013	Who assists during the labor?	01 Trained Dai<br>02 Untrained Dai<br>03 Doctor<br>04 Nurse<br>05 Others (specify)____________________
Q0014	Order of present birth (only live birth) *(include if the child died immediately after live birth)*	□□
Q0015	Did the baby cry immediately after birth?	01 Yes<br>02 NO

Q0016	When did you start breastfeeding?	01. Within first six hours<br>02. 6- 14 hours<br>03. 24- 48 hours<br>04. 48-72hours<br>05. After 72 hours of delivery<br>06. Never breastfeed so far (specify the reason)______________________
Q0017	Did you give colostrum to this child?	01 Yes<br>02 NO
Q0018	Did you feed anything else other than breastfeed to the baby in first 72 hours of birth?<br>If Yes, what is that	01 Yes<br>02 NO ____________________________________
Q0019	Weight of the child at the time of delivery?	☐☐☐☐ Grams<br>88 Don't Know
End time of the interview:☐☐:☐☐		
Checked by ____________________ Signature: _________ Date: ☐☐/☐☐/☐☐☐☐<br>Name of the data entry operator: ______________Signature : ______Date:☐☐/☐☐/☐☐☐☐		
